# ER-α36-Mediated Rapid Estrogen Signaling Positively Regulates ER-Positive Breast Cancer Stem/Progenitor Cells

**DOI:** 10.1371/journal.pone.0088034

**Published:** 2014-02-18

**Authors:** Hao Deng, Xin-Tian Zhang, Mo-Lin Wang, Hong-Yan Zheng, Li-Jiang Liu, Zhao-Yi Wang

**Affiliations:** 1 Departments of Medical Microbiology and Immunology, Creighton University Medical School, Omaha, Nebraska, United States of America; 2 Jiangda Pathology Center, Jianghan University, Wuhan, Hubei, P. R. China; 3 Institute of Medical Genetics, School of Medicine, Shandong University, Jinan, Shandong, P. R. China; University of South Alabama, United States of America

## Abstract

The breast cancer stem cells (BCSC) play important roles in breast cancer occurrence, recurrence and metastasis. However, the role of estrogen signaling, a signaling pathway important in development and progression of breast cancer, in regulation of BCSC has not been well established. Previously, we identified and cloned a variant of estrogen receptor α, ER-α36, with a molecular weight of 36 kDa. ER-α36 lacks both transactivation domains AF-1 and AF-2 of the 66 kDa full-length ER-α (ER-α66) and mediates rapid estrogen signaling to promote proliferation of breast cancer cells. In this study, we aim to investigate the function and the underlying mechanism of ER-α36-mediated rapid estrogen signaling in growth regulation of the ER-positive breast cancer stem/progenitor cells. ER-positive breast cancer cells MCF7 and T47D as well as the variants with different levels of ER-α36 expression were used. The effects of estrogen on BCSC's abilities of growth, self-renewal, differentiation and tumor-seeding were examined using tumorsphere formation, flow cytometry, indirect immunofluorence staining and *in vivo* xenograft assays. The underlying mechanisms were also studied with Western-blot analysis. We found that 17-β-estradiol (E2β) treatment increased the population of ER-positive breast cancer stem/progenitor cells while failed to do so in the cells with knocked-down levels of ER-α36 expression. Cells with forced expression of recombinant ER-α36, however, responded strongly to E2β treatment by increasing growth *in vitro* and tumor-seeding efficiency *in vivo*. The rapid estrogen signaling via the AKT/GSK3β pathway is involved in estrogen-stimulated growth of ER-positive breast cancer stem/progenitor cells. We concluded that ER-α36-mediated rapid estrogen signaling plays an important role in regulation and maintenance of ER-positive breast cancer stem/progenitor cells.

## Introduction

Accumulating experimental and clinical evidence supports that breast cancer may arise from mammary stem/progenitor cells that possess the ability to self-renew [1]–[4]. Al-Hajj et al, enriched a CD44^+^/CD24^−/low^ cell population from human breast cancer that displayed cancer stem/progenitor cell properties and was capable of forming tumors in immuno-compromised mice with higher efficiency than cells with alternative phenotypes [1]. Later, aldehyde dehydrogenase (ALDH) 1 expression and/or its activity were identified to be a marker for breast cancer stem/progenitor cells; fewer ALDH1 positive tumor cells than CD44^+^/CD24^−/low^ tumor cells were required to generate tumors *in vivo* [5]. The breast cancers with ALDH1^high^ cancer stem-like cells are often associated with more aggressive phenotypes such as estrogen receptor (ER) negativity, high histological grade, HER2 positivity, as well as poor prognosis [6].

Many signaling pathways involved in regulation of normal mammary stem cells including Hedgehog, Bmi-1, Wnt, NOTCH, HER-2, p53 and PTEN/Akt/β-catenin pathways play roles in breast cancer stem/progenitor cells [7]–[10]. However, the involvement of estrogen signaling, a major signaling pathway profoundly influences mammary carcinogenesis, in regulation of breast cancer stem/progenitor cells has not been well established, presumably since expression of estrogen receptor-α (ER-α) in breast cancer stem/progenitor cells remains controversial. It was reported that stem-like cells isolated from normal mammary gland and breast cancer tissues lack expression of the full-length ER-α [11], [12]. However, Clarke *et.al* reported that ER-α is expressed in putative normal breast stem/progenitor cells enriched by the “side population” method [13]. Despite the fact that ER expression in mammary stem cells is not clear, the significance of estrogen signaling for normal development and growth of the mammary gland is well established by studies in human and animal, which was explained as though indirect paracrine pathways [14]–[17].

Previously, we identified and cloned a novel variant of ER-α, which has a molecular weight of 36-kDa. Thus, we have named it ER-α36 [18], [19]. This ER-α variant differs from the original 66 kDa ER-α (ER-α66) because it lacks both transcriptional activation domains (AF-1 and AF-2) but retains the DNA-binding domain and partial ligand-binding domain [18]. It possesses a unique 27 amino acid stretch at the C-terminus to replace the last 138 amino acids of ER-α66. ER-α36 is mainly expressed at the plasma membrane and in the cytoplasm, and mediates non-genomic estrogen and antiestrogen signaling such as activation of the MAPK/ERK and PI3K/AKT signaling pathways [19], [20].

Using a specific anti-ER-α36 antibody, we previously found that ER-α36 is expressed in specimens from both ER-positive and –negative breast cancer patients [19], [21]–[23]. Recently, we reported that ER-α36-mediated estrogen signaling is critical for malignant growth of ER-negative breast cancer cells [24]. We also reported that ER-α36 expression is required for maintenance of the ALDH1-positive stem-like cells in ER-negative breast cancer SK-BR-3 cells [25], suggesting that ER-α36 is important in maintenance of the stem-like cells from ER-negative breast cancer. However, the function and underlying mechanisms of ER-α36-mediated estrogen signaling in regulation of the stem-like cells from ER-positive breast cancer are unknown.

Here, we show that ER-α36 is expressed in ER-positive breast cancer stem/progenitor cells, and ER-α36-mediated rapid estrogen signaling positively regulates ER-positive breast cancer stem/progenitor cells.

## Materials and Methods

### Reagents and Antibodies

The 17β-estradiol (E2β) was purchased from Sigma Chemical (St Louis, MO). The PI3K inhibitor LY294002 was from Tocris Bioscience (Ellisville, MO). The GSK-3β inhibitor IX, the AKT inhibitor IV, and the proteasome inhibitor MG132 were purchased from Calbiochem (San Diego, CA). The ER-α36 antibody was generated and characterized as described before [(19]. The β-actin antibody (1–19), anti-CK18 (DC-10) and anti-CD 10 (H-321) antibodies, anti-PCNA antibody (FL-261), the goat anti-mouse IgG-HRP, the goat anti-rabbit IgG-HRP and the donkey anti-goat IgG-HRP antibodies were purchased from Santa Cruz Biotechnology (Santa Cruz, CA). The ER-α antibody (ERAb-16) was purchased from NeoMarkers (Fremont, CA). The antibodies for AKT, p-AKT (Ser473), GSK-3β.27C∼_1_., p-GSK-3β.D85E12., β-Catenin (D10AB) and p-β-Catenin (thr41/Ser45) were all purchased from Cell Signaling Technology (Danvers, MA). The ALDH1 antibody (#61194) was from BD Biosciences (San Jose, CA). PerCP-Cy™5.5 mouse anti-human CD44 (clone C26) and PE mouse anti-human CD24 (clone ML5) were purchased from BD Pharmingen (San Jose, CA). Anti-rabbit Alexa Fluor 488 antibody (A-11008) and anti-mouse Alexa Fluor 555 antibody (A-31570) were from Invitrogen (Carlsbad, CA).

### Cell culture, Establishment of stable cell lines, and Growth assay

MCF7 and T47D cells were purchased from ATCC (Manassas, VA). The cells and their derivatives were cultured in Improved Minimal Essential Medium (IMEM) supplemented with 10% heat-inactivated fetal bovine serum (FBS), 1% non-essential amino-acids, 1% HEPES buffer, 1% antibiotic-antimycotic from Invitrogen (Carlsbad, CA) and 2 mg/ml bovine insulin (Sigma, St. Louis). All cells were maintained at 37°C and 5% CO2 in a humidified incubator.

MCF7 cells with forced expression of recombinant ER-α36 and with knocked-down levels of ER-α36 expression were established and characterized as described before [26], [27]. To establish stable cell lines with knocked-down expression of ER-α36 from T47D cells, we constructed an ER-α36 specific shRNA expression vector by cloning the DNA oligonucleotides 5′-GATGCCAATAGGTACTGAATTGATATCCGTTCAGTACCTATTGGCAT-3′ targeting the sequence in the 3′UTR of ER-α36 gene into the pRNAT-U6.1/Neo expression vector from GenScript Corp. Piscataway, NJ).

Briefly, T47D cells transfected with the empty expression vector and ER-α36 shRNA expression vector were selected with 500 µg/ml G418 for three weeks, and more than 20 individual clones from transfected cells were pooled, examined for ER-α36 expression with Western blot analysis and retained for experiments.

### Tumorsphere formation, Self-renewal and Growth assays

To establish tumorspheres, cells were seeded onto Corning Ultra-Low Attachment 6-well plate (Corning Incorporated, CA) at 10,000 cells/ml and cultured seven days in the tumorsphere medium: phenol-red free DMEM/F12 medium (Invitrogen) supplemented with 1× B-27 (Invitrogen), 20 ng/ml epidermal growth factor (Sigma-Aldrich) and 20 ng/ml basic fibroblast growth factor (ProSpec, NJ), 0.5 µg/mL hydrocortisone (Sigma). Tumorspheres were collected, washed with PBS, and incubated with Trypsin-EDTA (0.25%/0.5 mM) for two minutes at 37°C to dissociated cells, and cells were counted using the ADAM automatic cell counter (Digital Bio, Korea).

To assess the self-renewal of the stem-like cells, tumorspheres were dissociated and cell number was determined. The cells from 1^st^ generation of tumorspheres were seeded onto Ultra-Low Attachment 6-well plate at 5,000 cells/ml and cultured seven days in the tumorsphere medium to form 2^nd^ generation tumorspheres. The cells were then passed once a week for 3^rd^ and 4^th^ generation tumorspheres. The number of tumorspheres and dissociated cells were counted using a Multisizer 3 Coulter Counter (Beckman Coulter, Brea, CA) and the ADAM automatic cell counter, respectively. For estrogen stimulation assays, tumorspheres were treated with 0.1 nM E2β or vehicle (ethanol) as a control. Three dishes were used for each group and all experiments were repeated three times.

### Flow Cytometry Analysis

For CD44^+^/CD24^−^ cell analysis, single cell suspension washed with cold PBS/1% BSA were incubated with PerCP-Cy™5.5 mouse anti-human CD44 and PE mouse anti-human CD24 in PBS/1% BSA for 30 minutes at 4°C. After incubation, the cells were washed twice in cold PBS/1% BSA and re-suspended in cold PBS/1% BSA for flow cytometry analysis.

### DNA Transfection and Luciferase Assay

T47D and MCF7 cells were transfected with a p2×ERE-Luc reporter plasmid (a kind gift from Dr. Katarine Pettersson at Karolinska Institute, Sweden) using FuGene 6 transfection reagent (Roche Applied Science, Indianapolis, IN). Tumorspheres were transfected with electroporation using a pipette-type electroporator (MicroPorator MP-100, Digital Bio., Korean) as the manufacture recommended. All transfection included a cytomegalovirus-driven Renilla luciferase plasmid, pRL-CMV (Promega, Madison, WI) to establish transfection efficiency. Twenty-four hours after transfection, cells were treated with vehicle or 0.1 nM of E2β for 6, 12 and 24 hours. Cell extracts were prepared and luciferase activities were determined and normalized using the Dual-Luciferase Assay System (Promega, Madison, WI).

### Western Blot Analysis

Cells were washed with cold PBS and lysed with the RIPA buffer containing 1% proteinase inhibitor cocktail solution and 1% phosphatase inhibitor cocktail solution (Sigma). The cell lysates were boiled for 5 minutes in sodium dodecyl sulfate (SDS) gel-loading buffer and separated on 10% SDS-PAGE gels. After electrophoresis, the proteins were transferred to a polyvinylidene fluoride (PVDF) membrane (Bio-Rad Laboratories, Hercules, CA). The membranes were probed with appropriate primary antibodies and visualized with the corresponding secondary antibodies and the ECL kit (Thermo Scientific, Rockford, IL).

### Indirect Immunofluorescence Assay

Cells were fixed in 4% paraformaldehyde for 10 minutes, then permeabilized in 0.1% Triton X-100 for 5 minutes, blocked in 1% BSA for 30 minutes, and then incubated with primary antibodies at 4°C overnight. Secondary antibodies, anti-rabbit Alexa Fluor 488 or anti-mouse Alexa Fluor 555 were then added and incubated for 1 hour at room temperature. Cells were washed with PBS and mounted with 10 mg/ml DAPI (4,6-diamidino-2-phenylindole dihydrochloride) (Sigma-Aldrich) in aqueous mountant (Dako, Carpinteria, CA) and photographed using a fluorescent microscope (Nikon, Eclipss E600).

### Tumor Seeding Assays in Nude Mice

All animal procedures were approved by the Animal Care and Use Committee at the Creighton University and were performed in compliance with National Institutes of Health guidelines on the ethical use of animals. To assess tumor-seeding efficiency, cells in a serial dilution (1×10^2^, 1×10^3^, 1×10^4^ and 1×10^5^) were re-suspended in 0.1 ml of Matrigel and inoculated subcutaneously into the mammary fatpad of ovariectomized female nude mice (5–6 weeks old, strain CDI nu/nu, Charles River Breeding Laboratory). The mice were implanted with 0.35 mg/60-day slow-release 17β-estradiol pellets or placebos (Innovative Research of American, Sarasota, Florida) as controls. Mice were monitored twice a week for tumor growth. At the end of the experiments, the mice were euthanized, and the tumors were removed and weighed,

### Statistical Analysis

Data were summarized as the mean ± standard deviation (S.D.) using GraphPad InStat software program. Statistical analysis was performed using paired-samples t-test, or ANOVA followed by the Student–Newman–Keuls testing and the significance was accepted for P values less than 0.05.

## Results

### Estrogen Expands the Population of ER-positive Breast Cancer Stem/Progenitor Cells

**Figure 1:**
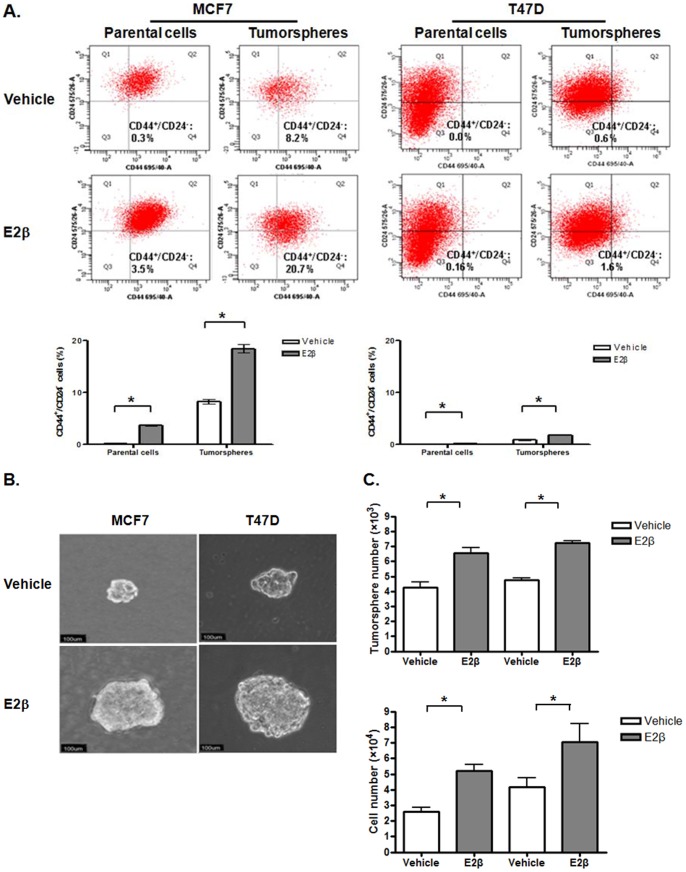
Estrogen expands the population of ER-positive breast cancer stem/progenitor cells. ER-positive breast cancer MCF7 and T47D cells were used. The tumorsphere formation assay and flow cytometry analysis of the CD44^−^ and CD24^+^ cells were used to assess the population of ER-positive breast cancer stem/progenitor cells. (A). Estrogen treatment increases the population of the CD44^−^/CD24^+^ cells in MCF7 and T47D cells. The monolayer (parental) and tumorspheres of MCF7 and T47D cells were treated with vehicle (ethanol) or 0.1 nM of E2β for five days. The population of CD44^−^/CD24^+^ cells in these cells were analyzed after staining with fluorochrome-conjugated antibodies. The representative results are shown on the upper panel. Lower panel: the columns represent the means of three experiments; bars, SE. *, P<0.05 for vehicle treated cells vs cells treated with E2β. (B). Estrogen treatment increases the size of tumorspheres from MCF7 and T47D cells. A representative tumorsphere from MCF7 and T47D cells treated with vehicle or 0.1 nM E2β for seven days. (C). Estrogen treatment increases the number of tumorspheres and cells from dissociated tumorspheres derived from MCF7 and T47D cells. The columns represent the means of three experiments; bars, SE. *, P<0.05 for cells treated with vehicle vs cells treated with E2β.

To examine the effects of estrogen signaling on growth of ER-positive breast cancer stem/progenitor cells, we used the well-characterized ER-positive breast cancer MCF7 and T47D cells as models. Both MCF7 and T47D cells were treated with or without 0.1 nM of E2β for five days. The CD44^+^/CD24^−^ stem-like cell populations in these cells were assessed with flow cytometry. We found that estrogen treatment significantly increased the CD44^+^/CD24^−^ cell population in both MCF7 and T47D cells (Figure 1A). We then cultured both MCF7 and T47D cells in the tumorsphere medium and under suspension conditions to form tumorspheres. We found that E2β treatment also increased the CD44^+^/CD24^−^ cell populations in tumorspheres from these cells (Figure 1A). We also found that E2β treatment markedly increased the size and number of the tumorspheres as well as the number of cells in the tumorspheres (Figure 1B and C). Our results thus suggested that estrogen signaling increases the population of ER-positive breast cancer stem/progenitor cells.

### ER-α36 Plays an Essential Role in Mitogenic Estrogen Signaling of ER-positive Breast Cancer Stem/Progenitor Cells

**Figure 2:**
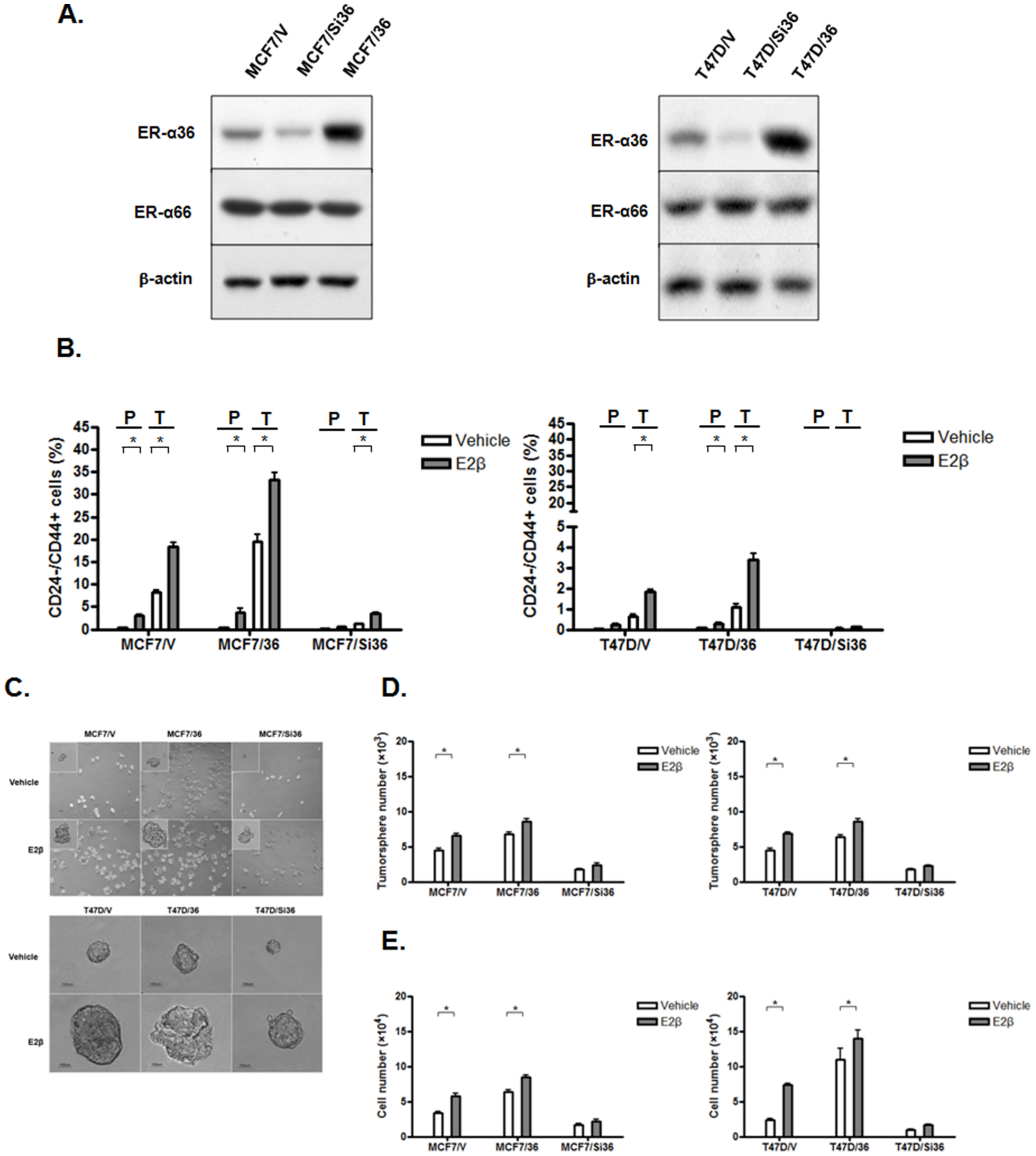
ER-α36-mediated rapid estrogen signaling positively regulates ER-positive breast cancer stem/progenitor cells. (A). Western blot analyses of ER-α36 expression in different MC7 and T47D cell variants; control cells (MCF7/V and T47D/V: cells transfected with the empty expression vector); cells with forced expression of ER-α36 (MCF7/36 and T47D/36: cells transfected with a ER-α36 expression vector); and ER-α36 expression knocked-down cells (MCF7/Si36 and T47D/Si36. (B). ER-α36-mediated estrogen signaling increases the population of the CD44^−^/CD24^+^ cells. The monolayer (parental, P) and tumorspheres (T) of MCF7 and T47D variants were treated with vehicle (ethanol) or 0.1 nM of E2β for five days. The population of CD44^−^/CD24^+^ cells in these cells were analyzed after staining with fluorochrome-conjugated antibodies. The columns represent the means of three experiments; bars, SE. *, P<0.05 for vehicle treated cells vs cells treated with E2β. (C). ER-α36-mediated estrogen signaling positively regulates the size and number of tumorspheres from MCF7 and T47D cells. Representative tumorspheres from MCF7 and T47D cell variants treated with vehicle or 0.1 nM E2β for seven days. Scale bar = 100 µm. (D). The numbers of tumorspheres and cells from dissociated tumorspheres of different cell variants were determined. The columns represent the means of three experiments; bars, SE. *, P<0.05 for cells treated with vehicle vs cells treated with E2β.

We then examined ER-α36 function in the stem/progenitor cells derived from ER-positive breast cancer cells. MCF7 and T47D cells transfected with the empty expression vector (MCF7/V and T47D/V), MCF7 and T47D cells with knocked-down levels of ER-α36 expression (MCF7/Si36 and T47D/Si36), and MCF7/36 and T47D/36 cells with high levels of recombinant ER-α36 expression were used (Figure 2A). The CD44^+^/CD24^−^ cell populations in parental MCF7 and T47D cells as well as different variants treated with or without E2β for five days were assessed. We found that in the MCF7 and T47D cells that express high levels of ER-α36, MCF7/36 and T47D/36, the populations of CD44^+^/CD24^−^ cells were significantly increased compared to the control cells transfected with the empty expression vector, suggesting that ER-α36 is involved in positive regulation of ER-positive breast cancer stem/progenitor cells (Figure 2B). Estrogen treatment further increased the populations of CD44^+^/CD24^−^ stem-like cells in MCF7/36 and T47D/36 cells (Figure 2B). We also examined the CD44^+^/CD24^−^ cell populations in the tumorspheres formed by these cells treated with or without E2β. We found that in the tumorspheres formed by MCF736 and T47D/36 cells, the populations of CD44^+^/CD24^−^ cells were dramatically increased compared to the control MCF7/V and T47D/V cells, which was further increased by estrogen treatment (Figure 2B). However, we found that the cells with knocked-down levels of ER-α36 expression, MCF7/Si36 and T47D/Si36, exhibited decreased populations of the CD44^+^/CD24^−^ cell and weakly responded to estrogen treatment (Figure 2B).

We then tested the capability of these cells to form tumorspheres. We found that in the absence of estrogen, the MCF7/36 and T47D/36 cells formed more and bigger tumorspheres compared to the control cells transfected with the empty expression vector (Figure 2C, D). Estrogen treatment further increased the number and size of tumorspheres formed by these cells (Figure 2C, D). The MCF7/Si36 and T47D/Si36 cells, however, formed less and smaller size tumorspheres compared to the control cells, and these cells responded poorly to estrogen stimulation (Figure 2C, D). We also collected tumorspheres, dissociated cells of the tumorspheres and assessed cell number. We found that in the MCF7 and T47D cells with knocked-down levels of ER-α36 expression, the cell numbers in tumorspheres were dramatically decreased compared to the control cells and were not increased in response to estrogen treatment (Figure 2E). On the other hand, in the MCF7 and T47D cells with forced expression of ER-α36, the number of cells in tumorspheres were significantly increased compared to the control cells and were further increased in response to estrogen treatment (Figure 2E). These results strongly indicated that the ER-positive breast cancer cells with high levels of ER-α36 expression contain higher percentage of stem/progenitor cells, and ER-α36 plays a critical role in estrogen-stimulated growth of ER-positive breast cancer stem/progenitor cells.

### ER-α36-mediated Estrogen Signaling Positively Regulates the Self-renewal of ER-positive Breast Cancer Stem Cells

**Figure 3:**
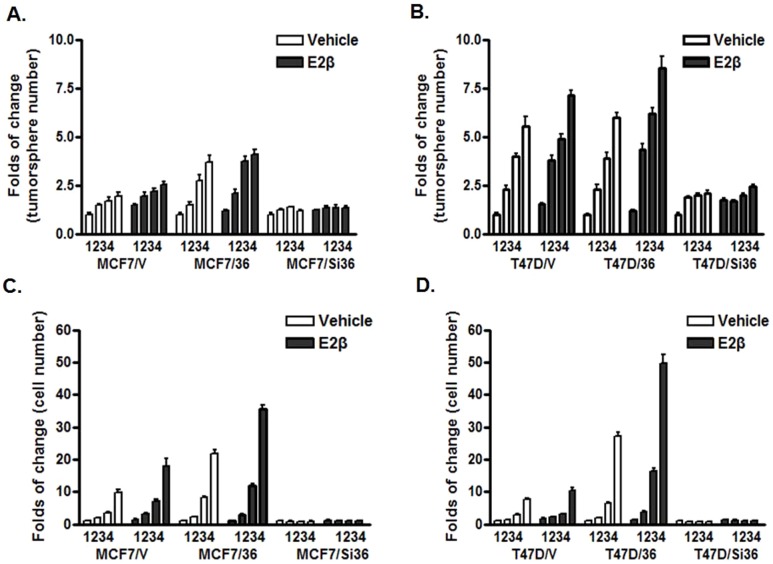
ER-α36-mediated estrogen signaling stimulates the self-renewal of ER-positive breast cancer stem cells. Long-term expansion of MCF7 (A.C) and T47D (B, D) variant cells in the presence of vehicle (ethanol) or 0.1 nM of E2β. The cells from tumorspheres were passed once a week for four generations. The numbers of tumorspheres and cells from dissociated tumorspheres were determined. The numbers of tumorspheres and cells from tumorspheres from the control cells transfected with the empty expression vector and treated with vehicle were arbitrarily set as 1. Three dishes were used for each group and the experiments were repeated three times. The columns represent the means of three experiments; bars, SE.

According to the stem cell model, stem cells divide asymmetrically to maintain homeostasis of the stem cell pool, a process called self-renewal, while the growth of the bulk population relies on progenitor cells. To examine whether ER-α36-mediated estrogen signaling also influences the self-renewal of ER-positive breast cancer stem cells, we studied the tumorsphere formation of MCF7 and T47D cells as well as their derivatives with different levels of ER-α36 expression through serial passages in the absence or presence of estrogen. The cells were treated with vehicle or E2β at the time of each seeding. All viable cells were determined at the end of each passage and seeded for next passage for a total of four passages. We found that the MCF7 and T47D control cells transfected with the empty expression vector produced more tumorspheres in 2^nd^, 3^rd^ and 4^th^ generations in the absence of estrogen while estrogen treatment further increased the number of tumorspheres in each generation (Figure 3A, B). Compared to the vector control cells, MCF7/36 and T47D/36 cells generated much more breast cancer stem/progenitor cells in 2^nd^, 3^rd^ and 4^th^ generations of the self-renewal in the absence of estrogen, and estrogen treatment further enhanced growth of these cells (Figure 3A, B). In the absence and presence of estrogen, MCF7/Si36 and T47D/Si36 failed to generate more tumorspheres in each generation (Figure 3A, B). We also dissociated tumorspheres and determined the cell number. We found that cell numbers were increased more dramatically than the tumorsphere number in both cell lines, especially in the presence of estrogen (Figure 3C, D). Our results thus suggested that ER-α36-mediated estrogen signaling positively regulates the self-renewal of ER-positive breast cancer stem cells.

### ER-α36-mediated Rapid Estrogen Signaling Enhances the Tumor-Seeding Efficiency of ER-positive Breast Cancer Stem/Progenitor Cells

**Figure 4:**
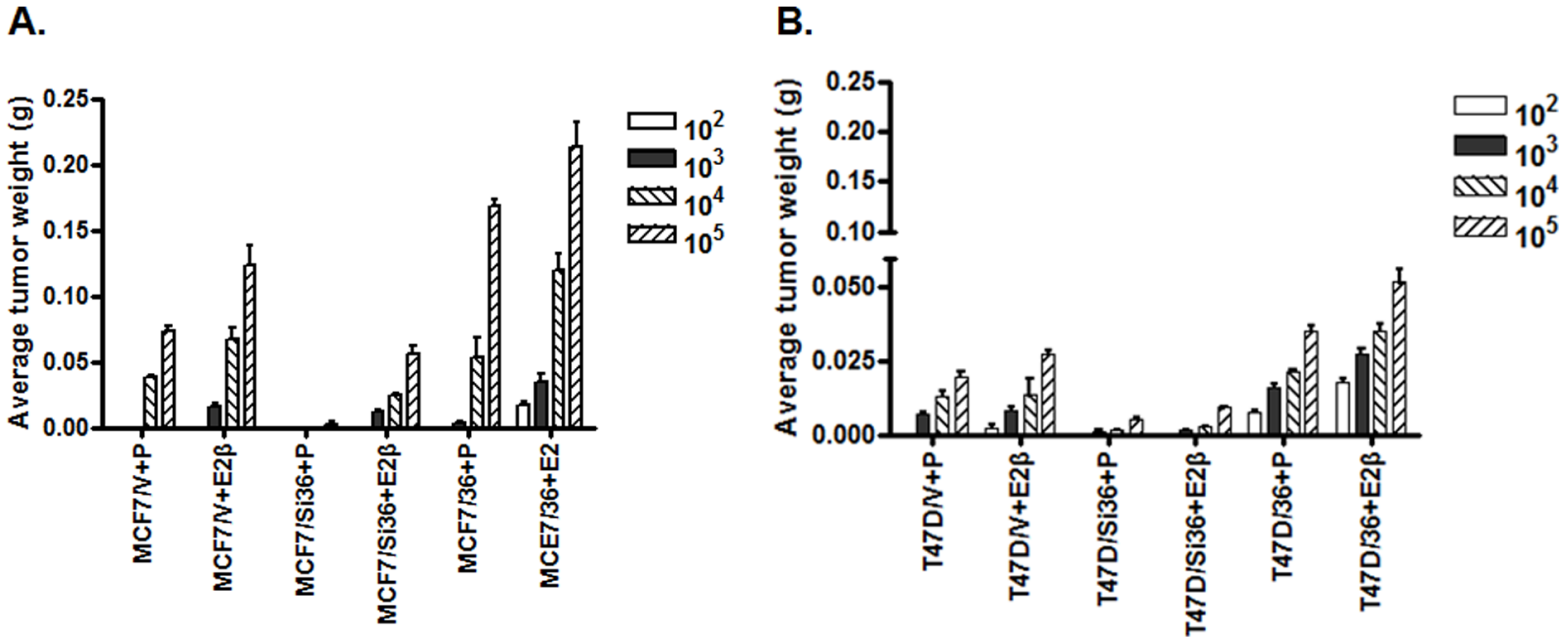
ER-α36-mediated estrogen signaling enhances the tumor-seeding efficiency of ER-positive breast cancer stem/progenitor cells. Different variants of MCF7 and T47D cells at limited dilutions were implanted in the mammary fatpad of the ovariectomized female mice supplemented with estrogen or placebo pellets. The tumor-seeding efficiency was examined by measurement of tumor weight. The data represent the mean ± SE observed in six mice in each group.

Previously, MCF7-derived tumorsphere cells were reported to be more tumorigenic than the parental cells [28]. To assess the involvement of ER-α36-mediated estrogen signaling in tumor seeding efficiency of ER-positive breast cancer stem/progenitor cells, we evaluated the tumor forming potential of tumorsphere cells derived from MCF7 and T47D cells and their variants with different levels of ER-α36 expression using *in vivo* tumorigenic assay. We first enriched the breast cancer stem/progenitor cells using the tumorsphere formation assay. The tumorsphere cells were then injected in serial limiting dilution (1×10^2^, 1×10^3^, 1×10^4^ and 1×10^5^ cells) into the mammary fatpad of ovariectomized female nude mice that were implanted with 0.35 mg/60-day slow-release 17β-estradiol or placebo pellets. In the absence of estrogen, tumorsphere cells from MCF7/V cells formed tumors at efficiency of four out of six mice and five out of six mice injected with 1×10^4^ and 1×10^5^ cells, respectively while MCF7/Si36 cells generated small tumors in four out of six mice only when 1×10^5^ cells was injected (Figure 4, Table S1). The tumorsphere cells from MCF7/36 cells, however, had high tumor initiating potential; forming tumors (5/6) at 1×10^3^ cells in the absence of estrogen. In the presence of estrogen, however, tumorsphere cells from MCF7/36 cells exhibited potent tumor-initiating efficiency, and generated tumors at 100 cells while MCF7V cells required 1,000 cells to generated tumors. We also found that MCF7/Si36 cells generated smaller tumors than the tumors formed by the control MCF7/V cells (Figure 4, Table S1). Similar results were also obtained in tumorsphere cells derived from T47D cell variants (Figure 4, Table S1). Our results thus strongly suggested that ER-α36-mediated estrogen signaling enhances the tumor-initiating efficiency of ER-positive breast cancer stem/progenitor cells.

### ER-α36-mediated Estrogen Signaling Induced Proliferation of Luminal Epithelial Lineage Specific ER-positive Breast Cancer Progenitor Cells

**Figure 5:**
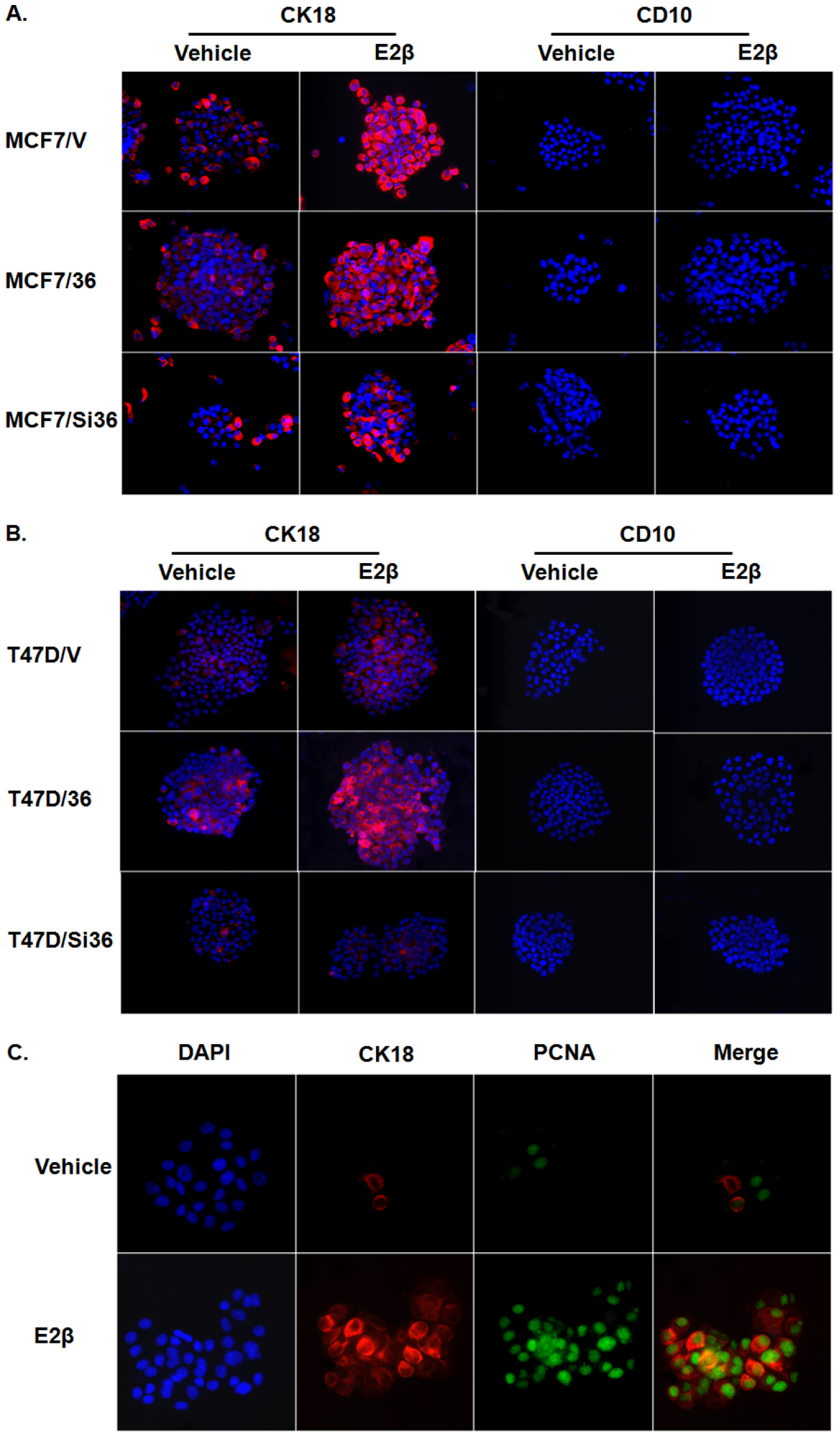
ER-α36-mediated estrogen signaling induced proliferation of luminal epithelial lineage specific ER-positive breast cancer progenitor cells. (A. B). Indirect Immunofluorescent staining for CK18 (red) or CD10 (red) in variants derived from MCF7 and T47D cells treated with vehicle or E2β. DAPI (blue) was used to stain the nuclear region. (C). Indirect Immunofluorescent staining for CK18 (red) or PCNA (green) in MCF7 cells treated with vehicle or E2β. DAPI (blue) was used to indicate the cell nuclei.

Breast cancer stem cells are able to differentiate into both luminal epithelial and myoepithelial cells [3]. We investigated the differentiation lineages of the stem cells derived from different MCF7 and T47D derivatives in the presence and absence of estrogen. Single cell suspensions from tumorspheres plated on collagen-coated coverslips or intact tumorspheres in suspension culture were treated with or without E2βfor five days, and the indirect immunofluoresces assay was performed to determine the effects of estrogen on differentiation lineages of these cells using cytokeratin 18 (CK18) for luminal epithelial cells and CD10 for myoepithelial cells. We found that tumorsphere cells plated on collagen-coated coverslips were fully differentiated into either luminal epithelial or myoepithelial lineages, and estrogen treatment had less or no effect on the differentiation (Figure S1), suggesting that estrogen treatment was unable to influence differentiation induced by cell attachment. We then assessed the effects of E2β on the spontaneous differentiation occurred in tumorspheres under suspension culture. In tumorspheres formed by MCF7 cells, we found that estrogen treatment increased the population of the cells that were stained positive for CK18 but without effect on the cells positive for CD10 (Figure 5A). We also found that estrogen treatment increased more number of cells expressing CK18 in MCF7/36 cells compared with MCF7/V cells (Figure 5A) while estrogen only slightly increased CK18 positive cells in MCF7/Si36 cells. Similar results were also observed in T47D cell variants; T47D/Si36 cells failed to respond to estrogen (Figure 5B).

To further examine whether estrogen treatment induces differentiation of breast cancer stem cells or increases proliferation of breast cancer progenitor cells that were in luminal epithelial lineage, we tested if the cells stained positive for CK18 were still proliferative. Indirect immunofluorescence staining was performed to examine the co-expression of CK18 and the proliferating cell nuclear antigen (PCNA), a marker for cell proliferation. We found that in MCF7 cells, the number of both PCNA and CK18 positive cells was low in the absence of estrogen. After estrogen treatment, however, the number of cells co-expressing both PCNA and CK18 was markedly increased (Figure 5C), indicating estrogen stimulates proliferation of luminal epithelial lineage specific breast cancer progenitor or intermediate cells.

### The PI3K/AKT/GSK3β/β-catenin Signaling Pathway is Involved in ER-α36-mediated Mitogenic Estrogen Signaling of ER-positive Breast Cancer Stem/Progenitor Cells

**Figure 6:**
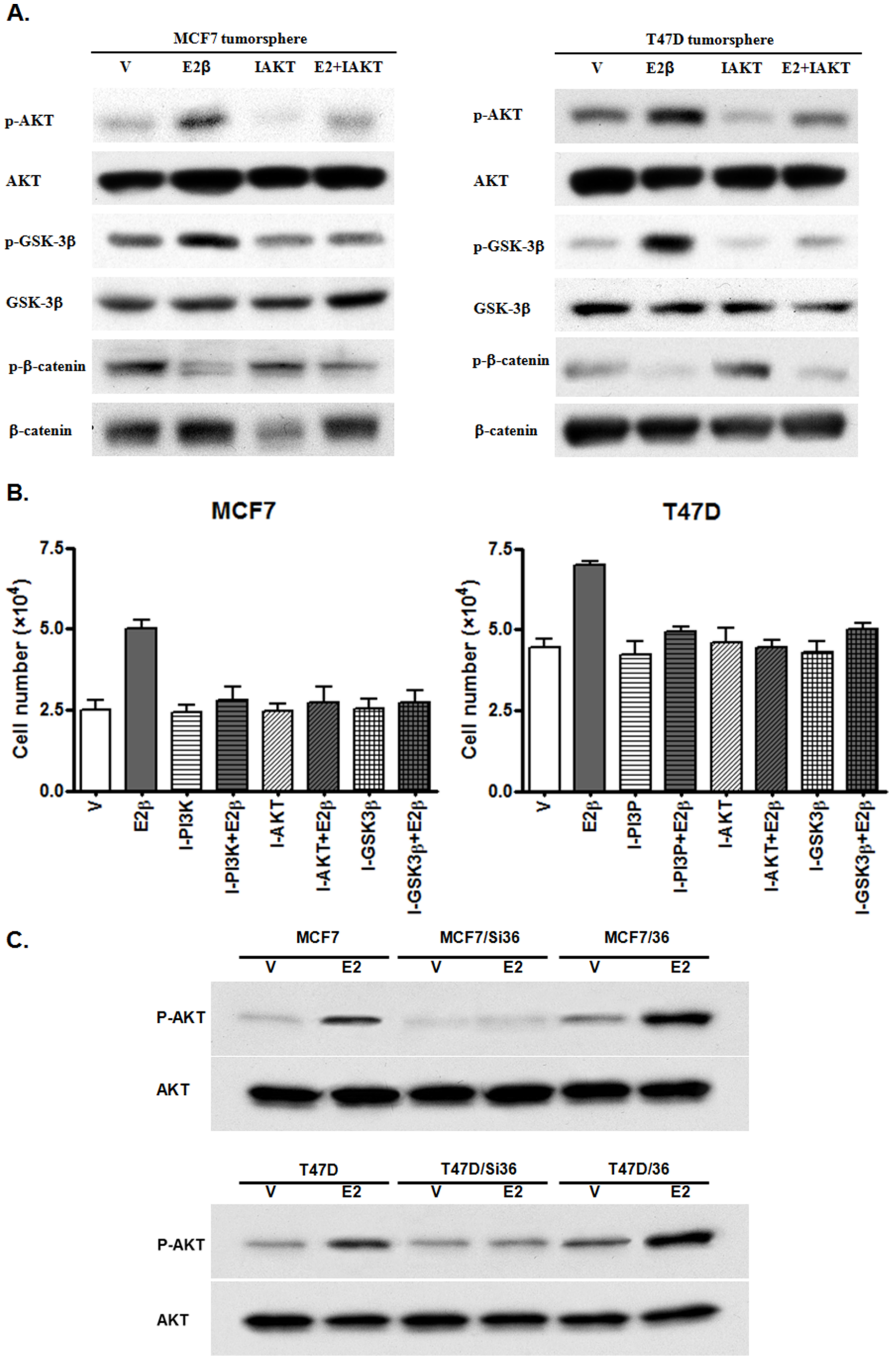
The PI3K/AKT/GSK3β/β-catenin signaling pathway is involved in ER-α36-mediated mitogenic estrogen signaling of ER-positive breast cancer stem/progenitor cells. (A). Western blot analysis of the cell lysates from tumorspheres derived from MCF7 and T47D cells treated with ethanol (V); 0.1 nM of E2β; the AKT inhibitor IV (10 µM), IAkt; and E2β+IAkt, using indicated antibodies. (B). The effects of different inhibitors of the PI3K/AKT/GSK3β pathway on estrogen-stimulated growth of ER-positive breast cancer stem/progenitor cells. Tumorspheres of MCF7 and T47D cells were treated with vehicle, E2β alone or E2β together with the PI3K inhibitor LY294002 (10 µM), the GSK-3β inhibitor IX (10 µM), the AKT inhibitor IV (10 µM). After seven days, cell numbers from dissociated tumorspheres were determined. The columns represent the means of three experiments; bars, SE.

We also investigated the underlying mechanism of ER-α36-mediated estrogen signaling in ER-positive breast cancer stem/progenitor cells. We treated tumorspheres formed by MCF7 and T47D cells with E2β and performed Western blot analysis using various phosphorylation specific for the AKT, GSK-3β and β-catenin. We found that estrogen induced the activation of the PI3K/AKT/GSK3β/β-catenin signaling pathway in ER-positive breast cancer stem/progenitor cells, which was attenuated by the AKT inhibitor (Figure 6A). We then included chemical inhibitors for the PI3K, AKT and GSK3β during estrogen stimulation and found that inhibition of the PI3K, AKT and GSK3β attenuated estrogen-stimulated growth of the stem/progenitor cells (Figure 6B). However, in the tumorspheres derived from MCF7/Si36 and T47D/Si36, estrogen failed to induce the AKT phosphorylation (Figure 6C). Our results thus indicated that the PI3K/AKT/GSK3β/β-catenin signaling pathway is involved in ER-α36-mediated mitogenic estrogen signaling of ER-positive breast cancer stem/progenitor cells.

### The Expression and Genomic Function of ER-α66 are Down-regulated in ER-positive Breast Cancer Stem/Progenitor Cells

Since the expression and potential function of ER-α66 in the breast cancer stem/progenitor cells remains controversial, we decided to study the expression pattern and possible function of ER-α66 in tumorsphere cells derived from MCF7 and T47D cells that express high levels of endogenous ER-α66.

**Figure 7:**
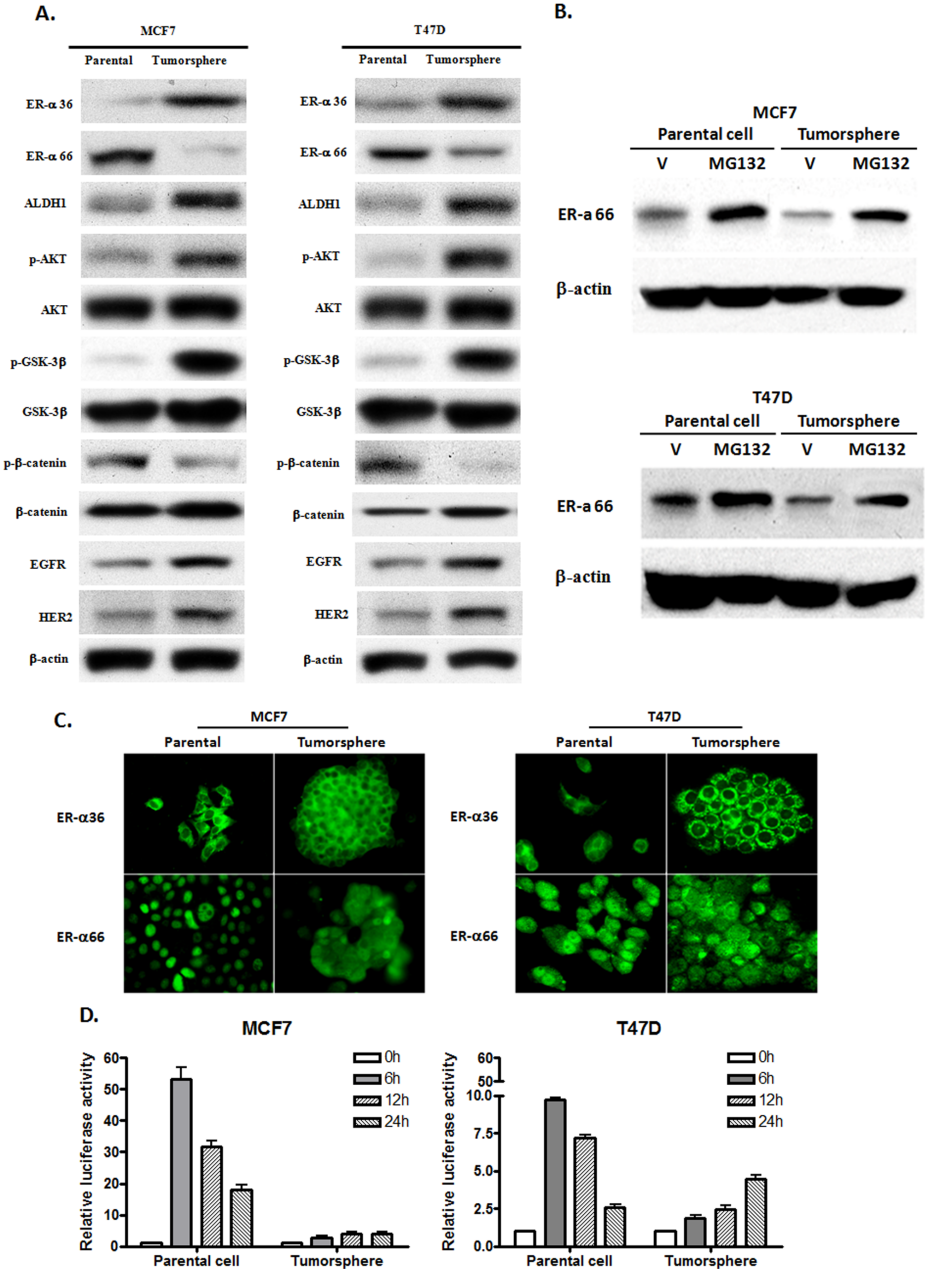
The expression and genomic function of ER-α66 are down-regulated in ER-positive breast cancer stem/progenitor cells. (A). Western blot analysis of the expression of different proteins in the monolayer cells (parental) and tumorspheres of the MCF7 and T47D cells. (B). Western blot analysis of ER-α66 expression in monolayer (parental) and tumorspheres of the MCF7 and T47D cells treated with or without the proteasome inhibitor MG132 (100 nM) for 12 hours. (C). Indirect Immunofluorescent staining for ER-α36 and ER-α66 in the monolayer cells (parental) and tumorspheres of the MCF7 and T47D cells. (D). The monolayer cells (parental) and tumorspheres of the MCF7 and T47D cells were transfected with the ERE luciferase report plasmid (2 µg). Twenty-four hours later, 0.1 nM of E2β was added and incubated for indicated time periods. The luciferase activities were assayed and normalized using a cytomegalovirus-driven Renilla luciferase plasmid. Two replicates were used in each experiment. Columns: means of four independent experiments; bars, SE.

To assess the expression levels of ER-α66 and ER-α36 in ER-positive breast cancer stem/progenitor cells, we performed Western blot analysis with cell lysates from tumorspheres. We found that the expression levels of ER-α36 protein were dramatically increased in tumorspheres from MCF7 and T47D cells while ER-α66 expression was down-regulated compared to parental cells (Figure 7A). In addition, we also found that the expression levels of ALDH1 and the basal levels of the AKT and GSK3β phosphorylation were markedly increased in tumorspheres (Figure 7A). The expression levels of growth receptors EGFR and HER2 were also increased in tumorspheres (Figure 7A). When the tumorspheres derived from MCF7 and T47D cells were treated with MG132, a proteasome inhibitor, the steady state level of ER-α66 protein was dramatically increased in both parental cells and tumorshphere cells (Figure 7B), suggesting that degradation of ER-α66 protein by the proteasome system is involved in regulation of the steady state levels of ER-α66, which was enhanced in ER-positive breast cancer stem/progenitor cells. We then examined the expression patterns of ER-α66 and 36 in parental and tumorsphere cells using the indirect immunofluorescence staining. We found that ER-α36 is expressed at the plasma membrane and in the cytoplasm of both parental and tumorsphere cells (Figure 7C). ER-α66, however, exhibited a predominant nuclear staining in the parental MCF7 and T47D cells while a weak cytoplasm staining was also observed in T47D cells. In tumorsphere cells, ER-α66 was mainly expressed in the cytoplasm (Figure 7C), indicating a great portion of ER-α66 protein was redistributed to the cytoplasms of ER-positive tumorsphere cells. When the parental MCF7 and T47D cells, and their tumorsphere cells were transfected with a ERE containing luciferase reporter plasmid and treated with or without estrogen, we found that estrogen-induced transcription activities of ER-α66 were dramatically reduced in tumorsphere cells compared to parental cells (Figure 7D), indicating the genomic estrogen signaling mediated by ER-α66 is attenuated in ER-positive breast cancer stem/progenitor cells.

The luminal compartment of mammary gland could be separated into ER-α66 positive and negative cells. The ER-α66 positive luminal cells express prolactin and progesterone receptor, and more luminal cytokeratins than ER-α66 negative luminal cells [11]. MCF7 cells were cultured in suspension culture for three and seven days to form tumorspheres. Indirect immunofluorescence staining was performed to examine the expression of CK18 and ER-α66 in cells from the tumorspheres. We found that in the cells cultured for three days, CK18 was highly expressed, and ER-α66 was expressed mainly in the cell nucleus (Figure S2). In the tumorspheres cultured for seven days, however, a great portion of ER-α66 was redistributed from the cell nucleus to the cytoplasm and the signals for CK18 was diminished (Figure S2), indicating a correlation between cytoplasmic distribution of ER-α66 and downregulation of cells expressing CK18. Taken together, our results strongly suggested that ER-α66-mediated genomic estrogen signaling is important in cell differentiation, which is attenuated in ER-positive breast cancer stem/progenitor cells presumably through re-distribution and down-regulation of ER-α66 protein.

### ER-α36 Expression is Positively Correlated to ALDH1 Expression in Specimens from Breast Cancer Patients

To further determine if ER-α36 is involved in positive regulation of breast cancer stem/progenitor cells *in vivo*, we examined the expression correlation of ER-α36 with ALDH1. We examined ER-α36 expression in sixty-eight specimens from breast cancer patients with the immunohistochemistry (IHC) assay and found that 34 out of 68 cases (50%) exhibited ER-α36 expression, predominantly in a cytoplasmic and membranous pattern (Figure S3, Table 1). The mean percentage of the ER-α36-positive cells was 53% and the majority of the cases showed moderate to strong ER-α36 staining. ALDH1 was detected in 30 cases (44%), 20 of which co-expressed ER-α36. There was a positive correlation between ER-α36 and ALDH1 expression (P<0.01, x^2^5.96). ER-α66 was expressed in 32 cases (47%), there was no correlation between ER-α66 and ALDH1 expression. These results suggested that ER-α36-mediated rapid estrogen signaling plays an important role in regulation of breast cancer stem/progenitor cells *in vivo*.

**Table 1:** The relationship between ER-α36, ER-α66 and ALDH1 in sixty-eight patients.

		ER-α36				ER-α66			
		+	−	*X* ^2^	P	+	−	*X* ^2^	P
ER-α66	+	19	13	2.13	>0.05	-	-	-	-
	−	15	21			-	-	-	-
ALDH1	+	20	10	5.96	<0.01	13	17	0.3	>0.05
	−	14	24			19	19		

The immunohistochemistry (IHC) assay was performed in specimens from sixty-eight patients. The results showed that ER-α36 had a positive correlation with ALDH1 (P<0.01, χ^2^5.96). There were no correlations between ER-α36 and ER-α66, and between ER-α66 and ALDH1.

## Discussion

In this study, the breast cancer stem/progenitor cells enriched from ER-positive breast cancer MCF7 and T47D cells were used as models to investigate their responses to estrogen. Here, we demonstrated that ER-α36-mediated rapid estrogen signaling plays an important role in maintenance and positive regulation of ER-positive breast cancer stem/progenitor cells. We showed that estrogen treatment expanded the population of breast cancer stem/progenitor cells and also stimulated the self-renewal of breast cancer stem cells, both of which were mediated by ER-α36. Knockdown of ER-α36 expression diminished tumor-seeding efficiency of ER-positive breast cancer stem/progenitor cells. We also showed that ER-α36 expression was markedly increased in the stem/progenitor cells enriched from ER-positive breast cancer cells accompanied by high levels of ALDH1, EGFR and HER2 as well as high levels of AKT and GSK3β phosphorylation. Finally, we presented evidence to indicate that the ER-α (ER-α66), was re-distributed outside of the cell nuclei, and its expression and genomic transcription activity were attenuated in ER-positive breast cancer stem/progenitor cells.

It is increasingly recognized that breast cancer has a population of cancer stem/progenitor cells that maintains tumor growth [29], [30]. However, the function and underlying mechanisms of estrogen signaling in regulation of breast cancer stem/progenitor cells are not clear. Mammary stem cells of human and mouse are highly responsive to estrogen signaling, although they usually show a receptor negative phenotype for ER-α and PR [31], [32]. A paracrine signaling model was proposed to explain the effects of estrogen signaling in mammary stem/progenitor cells [16], [17]. Here, we demonstrated, for the first time, that estrogen positively regulated ER-positive breast cancer stem/progenitor cells via ER-α36-mediated rapid signaling pathway.

Expression of the full-length ER-α66 in the stem-like cells isolated from normal mammary gland and breast cancer tissues is controversial [11]–[13]. Here, using the well-established ER-positive breast cancer cells, we demonstrated that ER-α66 protein was re-distributed from the cell nucleus to the cytoplasm and was destabilized presumably through the proteasome degradation system in ER-positive breast cancer stem/progenitor cells. As a result, the transcription activity of ER-α66 was attenuated in these cells. Thus, although ER-positive breast cancer stem/progenitor cells may retain ER-α66 expression, its function in genomic estrogen signaling may be diminished through redistribution and destabilization of the protein.

Previously, it was reported that ER-α66 positive luminal cells form a differentiated luminal compartment that express more luminal cytokeratins than ER-α66 negative luminal cells in mammary gland [17]. ER-α66 is often co-expressed with GATA3 in breast tumors and breast cancer cell lines [33]–[35]. GATA3 is a critical regulator of luminal differentiation that maintains the differentiation of the luminal cells in the mammary gland [36], [37]. Our finding here that redistribution and down-regulation of ER-α66 were associated with decreased number of cells positive for CK18 in tumorspheres from ER-positive breast cancer cells highlighted an important role of ER-α66 in differentiation of luminal epithelial cells.

Here, we found that estrogen treatment increased both the numbers and sizes of tumorspheres from the ER-positive breast cancer cells, suggesting estrogen treatment expanded the pool of ER-positive breast cancer stem/progenitor cells via ER-α36-mediated signaling. Stem cells maintain self-renewal and differentiation in two ways: asymmetric and symmetric cell division [38], [39]. Accumulating evidence suggested that dysregulation of asymmetric stem cell division is one of the reason for stem cell transformation [38], [39]. However, the mechanisms by which stem cells adapt symmetric division have not been fully understood. Cicalese et al. reported that breast cancer stem cells derived from ERBB2/HER2 transgenic mice exhibited an increased frequency of symmetric self-renewing cell divisions and implicated p53 is a master regulator of this process [40]. Here, we found that in the presence of estrogen, ER-positive breast cancer cells with forced expression of ER-α36 increased the populations of breast cancer stem cells as evidenced by increased sizes and numbers of tumorspheres formed by these cells. However, cells with knocked-down levels of ER-α36 expression failed to increase the populations of stem/progenitor cells in response to estrogen while still retained the ability of the self-renewal. Since there are no specific markers to differentiate breast cancer stem, progenitor, and intermediate cells (non-stem proliferative cells), it is difficult to determine which cell populations that estrogen stimulates. However, the results that estrogen treatment increased both size and number of tumorspheres formed by ER-positive breast cancer cells and CK18 positive cells still underwent estrogen-stimulated cell proliferation suggested that ER-α36-mediated estrogen signaling may stimulate proliferation of breast cancer stem, progenitor and intermediate cells, and also suggested that ER-α36 overexpression might be involved in symmetric stem cell division.

The genomic or classic estrogen-signaling pathway mediated by ER-α66 is prevailingly thought to be responsible for the initiation and progression of breast cancer. However, we found that knock-down of ER-α36 expression in the ER-positive breast cancer cells diminished the tumor-seeding efficiency of the breast cancer stem/progenitor cells and the genomic estrogen-signaling mediated by ER-α66 is attenuated in the ER-positive breast cancer stem/progenitor cells. Additionally, the nuclear expression of ER-α66 is correlated with differentiation of luminal epithelial cells. Our results are in good agreement with a recent report that knock-down of ER-α66 expression in MCF7 cells using the shRNA method was without effect on tumorsphere formation and tumor-seeding potential in nude mice [41]. Together, these results suggested that ER-α36-mediated rapid estrogen signaling plays an important role in maintenance and regulation of ER-positive breast cancer stem/progenitor cells while ER-α66-mediated genomic estrogen signaling is involved in determination of luminal epithelial lineage specific differentiation.

Recently, we reported that ER-positive breast cancer cells expressing high levels of ER-α36 are more resistant to antiestrogen tamoxifen [27], consistent with our previous report that the breast cancer patients with tumors expressing high levels of ER-α36 less benefited from tamoxifen therapy compared to those with low levels of ER-α36 expression, and ER-α36 expression is significantly associated with HER2 expression [22], suggesting that increased ER-α36 expression is one of the underlying mechanisms of tamoxifen resistance. Here, we found that ER-α36 is highly expressed in ER-positive breast cancer stem/progenitor cells and plays an important role in positive regulation of these cells. Taken together, our results suggest that ER-positive breast cancer stem/progenitor cells may be resistant to antiestrogen tamoxifen.

In summary, our results provided strong evidence to support an important role of ER-α36-mediated rapid estrogen signaling in maintenance and regulation of ER-positive breast cancer stem/progenitor cells and provided a rational for development of therapeutic approaches to restrict growth of breast cancer stem/progenitor cells by targeting ER-α36.

## Materials and Methods

### Reagents, and Antibodies

The 17β-estradiol (E2β) was purchased from Sigma Chemical (St Louis, MO). The PI3K inhibitor LY294002 was from Tocris Bioscience (Ellisville, MO). The GSK-3β inhibitor IX, the AKT inhibitor IV, and the proteasome inhibitor MG132 were purchased from Calbiochem (San Diego, CA). The ER-α36 antibody was generated and characterized as described before [19]. The β-actin antibody (1–19), the goat anti-mouse IgG-HRP, the goat anti-rabbit IgG-HRP and the do nkey anti-goat IgG-HRP antibodies were purchased from Santa Cruz Biotechnology (Santa Cruz, CA). The ER-α antibody (ERAb-16) was purchased from NeoMarkers (Fremont, CA). The antibodies for AKT (#9772), p-AKT (Ser473, #9271), GSK-3β.clone.7., p-GSK-3β.Y216/, β-Catenin (clone 14) and p-β-Catenin (Ser33/37/Thr41, #9561) were purchased from Cell Signaling Technology (Danvers, MA). The ALDH1 antibody (#61194) was from BD Biosciences (San Jose, CA). PerCP-Cy™5.5 mouse anti-human CD44 (clone C26) and PE mouse anti-human CD24 (clone ML5) were purchased from BD Pharmingen (San Jose, CA). Anti-rabbit Alexa Fluor 488 antibody (A-11008) and anti-mouse Alexa Fluor 555 antibody (A-31570) were from Invitrogen (Carlsbad, CA).

### Cell culture, Establishment of stable cell lines, and Growth assay

MCF7 and T47D cells were purchased from ATCC (Manassas, VA). The cells and their derivatives were cultured in Improved Minimal Essential Medium (IMEM) supplemented with 10% heat-inactivated fetal bovine serum (FBS), 1% non-essential amino-acids, 1% HEPES buffer, 1% antibiotic-antimycotic from Invitrogen (Carlsbad, CA) and 2 mg/ml bovine insulin (Sigma, St. Louis). All cells were maintained at 37°C and 5% CO2 in a humidified incubator.

MCF7 cells with forced expression of recombinant ER-α36 and with knocked-down levels of ER-α36 expression were established and characterized as described before [26], [27]. To establish stable cell lines with knocked-down expression of ER-α36 from T47D cells, we constructed an ER-α36 specific shRNA expression vector by cloning the DNA oligonucleotides 5′-GATGCCAATAGGTACTGAATTGATATCCGTTCAGTACCTATTGGCAT-3′ targeting the sequence in the 3′UTR of ER-α36 gene into the pRNAT-U6.1/Neo expression vector from GenScript Corp. Piscataway, NJ).

Briefly, T47D cells transfected with the empty expression vector and ER-α36 shRNA expression vector were selected with 500 µg/ml G418 for three weeks, and more than 20 individual clones from transfected cells were pooled, examined for ER-α36 expression with Western blot analysis and retained for experiments.

### Tumorsphere formation, Self-renewal and Growth assays

To establish tumorspheres, cells were seeded onto Corning Ultra-Low Attachment 6-well plate (Corning Incorporated, CA) at 10,000 cells/ml and cultured seven days in the tumorsphere medium: phenol-red free DMEM/F12 medium (Invitrogen) supplemented with 1× B-27 (Invitrogen), 20 ng/ml epidermal growth factor (Sigma-Aldrich) and 20 ng/ml basic fibroblast growth factor (ProSpec, NJ), 0.5 µg/mL hydrocortisone (Sigma). Tumorspheres were collected, washed with PBS, and incubated with Trypsin-EDTA (0.25%/0.5 mM) for two minutes at 37°C to dissociated cells, and cells were counted using the ADAM automatic cell counter (Digital Bio, Korea).

To assess the self-renewal of the stem-like cells, tumorspheres were dissociated and cell number was determined. The cells from 1^st^ generation of tumorspheres were seeded onto Ultra-Low Attachment 6-well plate at 5,000 cells/ml and cultured seven days in the tumorsphere medium to form 2^nd^ generation tumorspheres. The cells were then passed once a week for 3^rd^ and 4^th^ generation tumorspheres. The number of tumorspheres and dissociated cells were counted using a Multisizer 3 Coulter Counter (Beckman Coulter, Brea, CA) and the ADAM automatic cell counter, respectively. For estrogen stimulation assays, tumorspheres were treated with 0.1 nM E2β or vehicle (ethanol) as a control. Three dishes were used for each group and all experiments were repeated three times.

### Flow Cytometry Analysis

For CD44^+^/CD24^−^ cell analysis, single cell suspension washed with cold PBS/1% BSA were incubated with PerCP-Cy™5.5 mouse anti-human CD44 and PE mouse anti-human CD24 in PBS/1% BSA for 30 minutes at 4°C. After incubation, the cells were washed twice in cold PBS/1% BSA and re-suspended in cold PBS/1% BSA for flow cytometry analysis.

### DNA Transfection and Luciferase Assay

T47D and MCF7 cells were transfected with a p2×ERE-Luc reporter plasmid (a kind gift from Dr. Katarine Pettersson at Karolinska Institute, Sweden) using FuGene 6 transfection reagent (Roche Applied Science, Indianapolis, IN). Tumorspheres were transfected with electroporation using a pipette-type electroporator (MicroPorator MP-100, Digital Bio., Korean) as the manufacture recommended. All transfection included a cytomegalovirus-driven Renilla luciferase plasmid, pRL-CMV (Promega, Madison, WI) to establish transfection efficiency. Twenty-four hours after transfection, cells were treated with vehicle or 0.1 nM of E2β for 6, 12 and 24 hours. Cell extracts were prepared and luciferase activities were determined and normalized using the Dual-Luciferase Assay System (Promega, Madison, WI).

### Western Blot Analysis

Cells were washed with cold PBS and lysed with the RIPA buffer containing 1% proteinase inhibitor cocktail solution and 1% phosphatase inhibitor cocktail solution (Sigma). The cell lysates were boiled for 5 minutes in sodium dodecyl sulfate (SDS) gel-loading buffer and separated on 10% SDS-PAGE gels. After electrophoresis, the proteins were transferred to a polyvinylidene fluoride (PVDF) membrane (Bio-Rad Laboratories, Hercules, CA). The membranes were probed with appropriate primary antibodies and visualized with the corresponding secondary antibodies and the ECL kit (Thermo Scientific, Rockford, IL).

### Indirect Immunofluorescence Assay

Cells were fixed in 4% paraformaldehyde for 10 minutes, then permeabilized in 0.1% Triton X-100 for 5 minutes, blocked in 1% BSA for 30 minutes, and then incubated with primary antibodies at 4°C overnight. Secondary antibodies, anti-rabbit Alexa Fluor 488 or anti-mouse Alexa Fluor 555 were then added and incubated for 1 hour at room temperature. Cells were washed with PBS and mounted with 10 mg/ml DAPI (4,6-diamidino-2-phenylindole dihydrochloride) (Sigma-Aldrich) in aqueous mountant (Dako, Carpinteria, CA) and photographed using a fluorescent microscope (Nikon, Eclipss E600).

### Tumor Seeding Assays in Nude Mice

All animal procedures were approved by the Animal Care and Use Committee at the Creighton University and were performed in compliance with National Institutes of Health guidelines on the ethical use of animals. To assess tumor-seeding efficiency, cells in a serial dilution (1×10^2^, 1×10^3^, 1×10^4^ and 1×10^5^ cells) were re-suspended in 0.1 ml of Matrigel and inoculated subcutaneously into the mammary fatpad of ovariectomized female nude mice (5–6 weeks old, strain CDI nu/nu, Charles River Breeding Laboratory). The mice were implanted with 0.35 mg/60-day slow-release 17β-estradiol pellets or placebos (Innovative Research of American, Sarasota, Florida) as controls. Mice were monitored twice a week for tumor growth. At the end of the experiments, the mice were euthanized, and the tumors were removed and weighed.

### Statistical Analysis

Data were summarized as the mean ± standard deviation (S.D.) using GraphPad InStat software program. Statistical analysis was performed using paired-samples t-test, or ANOVA followed by the Student–Newman–Keuls testing and the significance was accepted for P values less than 0.05.

## Supporting Information

Figure S1 **Estrogen failed to influence differentiation of ER-positive breast cancer stem cells cultured on collagen-coated coverslips.** The putative stem cells from tumorspheres derived from variants of ER-positive breast cancer MCF7 (A) and T47D (B) cells were cultured on collagen-coated coverslips for five days in the presence of vehicle or 0.1 nM E2β. Indirect Immunofluorescent staining for CD10 (red) and CK18 (red) in the cells. DAPI (blue) indicates the cell nuclei.
(TIF)

Figure S2 **Nuclear ER-α66 expression is correlated to CK18 expression in tumorspheres from MCF7 cells.** (A). Indirect Immunofluorescent staining for ER-α66 (green) and CK18 (red) in the tumorspheres of the MCF7 cells cultured for three and seven days. DAPI (blue) indicates the cell nuclei.
(TIF)

Figure S3 **Immunohistochemical staining of ALDH1, ER-α36 and ER-α66 in a breast cancer specimen.** Tissue from one patient showing strong, cytoplasmic and membrane expression of ALDH1 (A) and ER-α36 (B) but no ER-α66 expression (C) (all at ×400 magnification).
(TIF)

Table S1 **Summary of tumor formation assay.** The ovariectomized female nude mice (5–6 weeks old, strain CDI nu/nu) were implanted with 0.35 mg/60-day slow-release 17β-estradiol pellets or placebos as controls five days before tumor cell injection; n = six mice per group. Tumor cells as indicated in a serial dilution (1×10^2^, 1×10^3^, 1×10^4^ and 1×10^5^) were re-suspended in 100 µl of Matrigel and inoculated subcutaneously into the mammary fatpads of nude mice (one tumor per mouse). Tumors from MCF7 variants were harvested at 42 days and T47D variants at 40 days.
(DOCX)
