# Supplementary figures and images for: ER-α36-Mediated Rapid Estrogen Signaling Positively Regulates ER-Positive Breast Cancer Stem/Progenitor Cells
Source: PLoS One. 2014 Feb 18;9(2):e88034. doi: 10.1371/journal.pone.0088034 (PMC3928099; doi:10.1371/journal.pone.0088034)

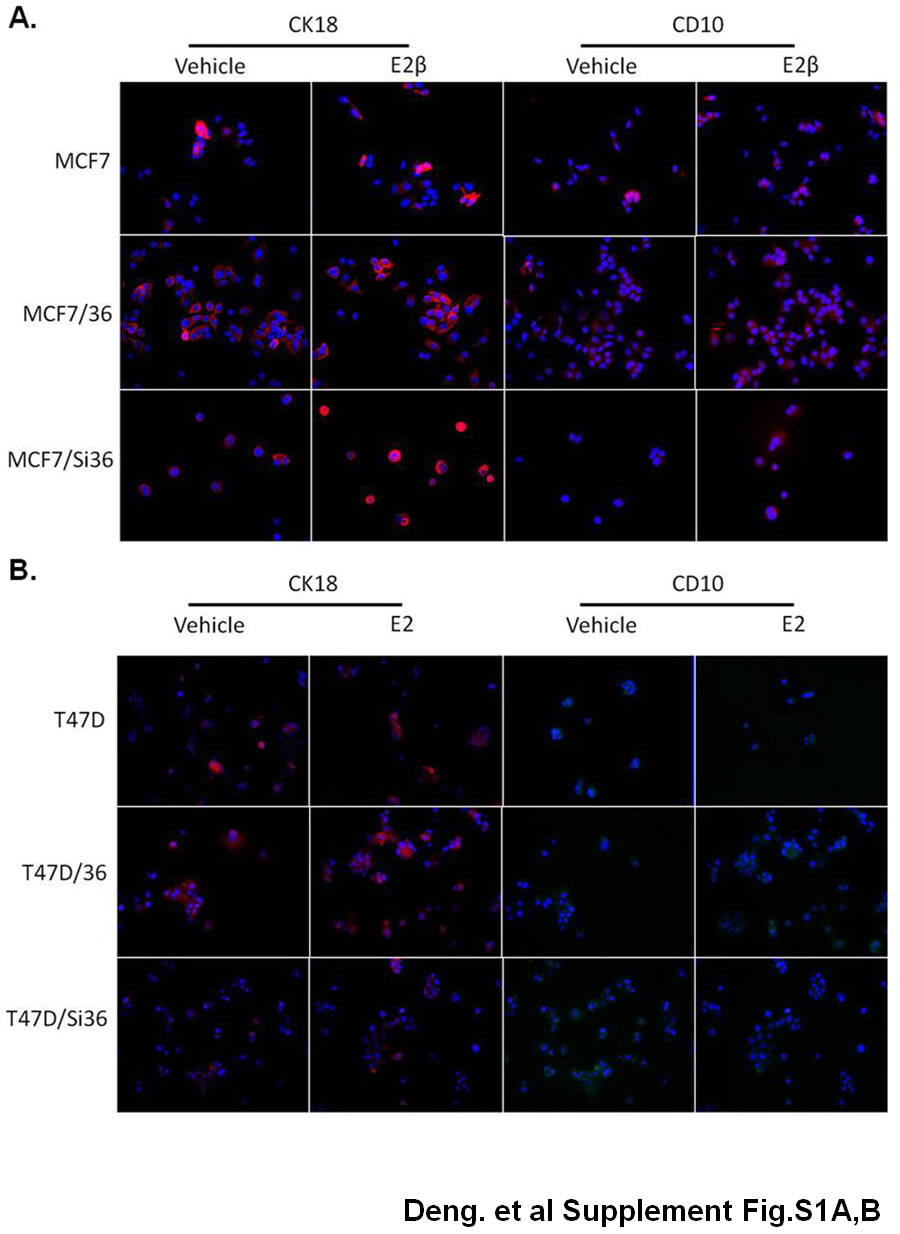

Supplement: Figure S1 — Estrogen failed to influence differentiation of ER-positive breast cancer stem cells cultured on collagen-coated coverslips. The putative stem cells from tumorspheres derived from variants of ER-positive breast cancer MCF7 (A) and T47D (B) cells were cultured on collagen-coated coverslips for five days in the presence of vehicle or 0.1 nM E2β. Indirect Immunofluorescent staining for CD10 (red) and CK18 (red) in the cells. DAPI (blue) indicates the cell nuclei. (TIF) [file pone.0088034.s001.tif]

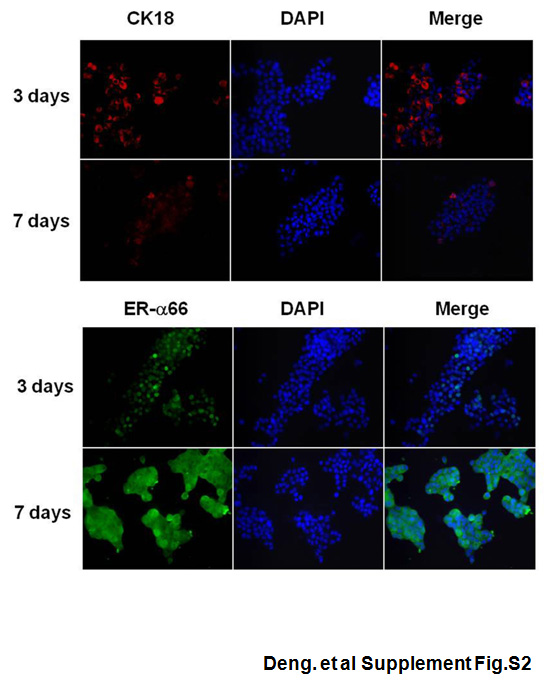

Supplement: Figure S2 — Nuclear ER-α66 expression is correlated to CK18 expression in tumorspheres from MCF7 cells. (A). Indirect Immunofluorescent staining for ER-α66 (green) and CK18 (red) in the tumorspheres of the MCF7 cells cultured for three and seven days. DAPI (blue) indicates the cell nuclei. (TIF) [file pone.0088034.s002.tif]

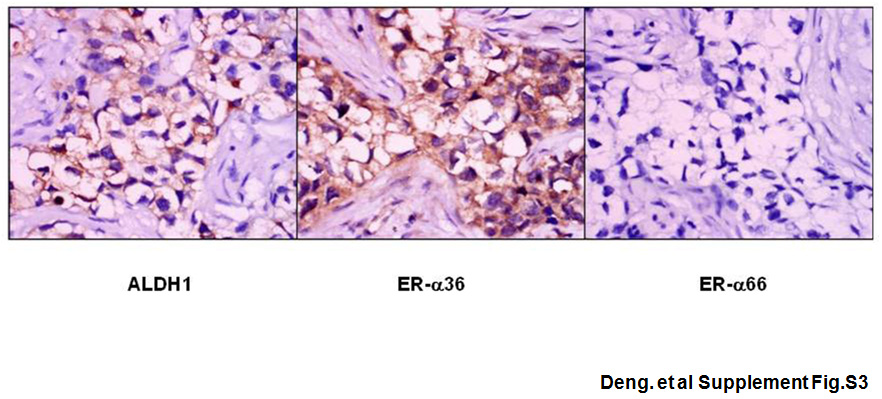

Supplement: Figure S3 — Immunohistochemical staining of ALDH1, ER-α36 and ER-α66 in a breast cancer specimen. Tissue from one patient showing strong, cytoplasmic and membrane expression of ALDH1 (A) and ER-α36 (B) but no ER-α66 expression (C) (all at ×400 magnification). (TIF) [file pone.0088034.s003.tif]
