# Supplementary material for: ER-α36-Mediated Rapid Estrogen Signaling Positively Regulates ER-Positive Breast Cancer Stem/Progenitor Cells
Source: PLoS One. 2014 Feb 18;9(2):e88034. doi: 10.1371/journal.pone.0088034 (PMC3928099; doi:10.1371/journal.pone.0088034)
Supplement: Table S1 — Summary of tumor formation assay. The ovariectomized female nude mice (5–6 weeks old, strain CDI nu/nu) were implanted with 0.35 mg/60-day slow-release 17β-estradiol pellets or placebos as controls five days before tumor cell injection; n = six mice per group. Tumor cells as indicated in a serial dilution (1×10^2^, 1×10^3^, 1×10^4^ and 1×10^5^) were re-suspended in 100 µl of Matrigel and inoculated subcutaneously into the mammary fatpads of nude mice (one tumor per mouse). Tumors from MCF7 variants were harvested at 42 days and T47D variants at 40 days. (DOCX) [file pone.0088034.s004.docx]

**Supplement Table S1**. Summary of tumor formation assay.

The ovariectomized female nude mice (5-6 weeks old, strain CDI nu/nu) were implanted with 0.35mg/60-day slow-release 17β-estradiol pellets or placebos as controls five days before tumor cell injection; n= six mice per group. Tumor cells as indicated in a serial dilution (1 × 10^2^, 1 × 10^3^, 1× 10^4^ and 1 × 10^5^) were re-suspended in 100 μl of Matrigel and inoculated subcutaneously into the mammary fatpads of nude mice (one tumor per mouse). Tumors from MCF7 variants were harvested at 42 days and T47D variants at 40 days.

|  | MCF7 tumorsphere | | | | T47D tumorsphere | | | |
| --- | --- | --- | --- | --- | --- | --- | --- | --- |
|  | placebo | | E2β | | placebo | | E2β | |
| Transfection | # of cells injected | Tumor seeding efficiency | # of cells injected | Tumor seeding efficiency | # of cells injected | Tumor seeding efficiency | # of cells injected | Tumor seeding efficiency |
| V | 1×10^2^ | 0/6 | 1×10^2^ | 0/6 | 1×10^2^ | 0/6 | 1×10^2^ | 4/6 |
|  | 1×10^3^ | 0/6 | 1×10^3^ | 6/6 | 1×10^3^ | 4/6 | 1×10^3^ | 6/6 |
|  | 1×10^4^ | 4/6 | 1×10^4^ | 6/6 | 1×10^4^ | 5/6 | 1×10^4^ | 6/6 |
|  | 1×10^5^ | 5/6 | 1×10^5^ | 6/6 | 1×10^5^ | 5/6 | 1×10^5^ | 6/6 |
| 36 | 1×10^2^ | 0/6 | 1×10^2^ | 6/6 | 1×10^2^ | 6/6 | 1×10^2^ | 6/6 |
|  | 1×10^3^ | 5/6 | 1×10^3^ | 6/6 | 1×10^3^ | 6/6 | 1×10^3^ | 6/6 |
|  | 1×10^4^ | 5/6 | 1×10^4^ | 6/6 | 1×10^4^ | 6/6 | 1×10^4^ | 6/6 |
|  | 1×10^5^ | 6/6 | 1×10^5^ | 6/6 | 1×10^5^ | 6/6 | 1×10^5^ | 6/6 |
| Si36 | 1×10^2^ | 0/6 | 1×10^2^ | 0/6 | 1×10^2^ | 0/6 | 1×10^2^ | 0/6 |
|  | 1×10^3^ | 0/6 | 1×10^3^ | 2/6 | 1×10^3^ | 0/6 | 1×10^3^ | 3/6 |
|  | 1×10^4^ | 0/6 | 1×10^4^ | 4/6 | 1×10^4^ | 3/6 | 1×10^4^ | 5/6 |
|  | 1×10^5^ | 4/6 | 1×10^5^ | 6/6 | 1×10^5^ | 5/6 | 1×10^5^ | 6/6 |
